# Antifungal Activity of Thai Cajuput Oil and Its Effect on Efflux-Pump Gene Expression in Fluconazole-Resistant *Candida albicans* Clinical Isolates

**DOI:** 10.1155/2020/5989206

**Published:** 2020-11-04

**Authors:** Pitchayaphong Keereedach, Karnjana Hrimpeng, Khaemaporn Boonbumrung

**Affiliations:** ^1^Department of Transfusion Medicine and Clinical Microbiology, Faculty of Allied Health Sciences, Chulalongkorn University, Bangkok 10330, Thailand; ^2^Department of Microbiology, Faculty of Science, Burapha University, Chonburi 20131, Thailand

## Abstract

Candidiasis caused by the fluconazole-resistant opportunistic pathogen *Candida albicans* is an intractable clinical problem that threatens immunocompromised or normal individuals. The most common mechanism of fluconazole resistance in *C. albicans* is the failure of cells to accumulate the drug due to increased expression of the efflux proteins encoded by the *CDR*1, *CDR*2, and *MDR*1 genes. Because the number of current antifungal drugs is limited, it is necessary to develop new therapeutic strategies. This study aimed to evaluate the antifungal activity of Thai Cajuput oil, its synergism with fluconazole, and its effect on efflux-pump gene expression in fluconazole-resistant *C. albicans* clinical isolates. Thus, we first detected the efflux-pump genes in fourteen resistant strains by PCR. The frequencies of the *CDR*1, *CDR*2, and *MDR*1 genes were 68.75%, 62.5%, and 87.5%, respectively, and these efflux-pump genes were distributed in three distinct patterns. Subsequently, the antifungal activity of Thai Cajuput oil was assessed by broth macrodilution and its synergism with fluconazole was evaluated by the checkerboard assay. The changes in the expression levels of *CDR*1, *CDR*2, and *MDR*1 after treatment with Thai Cajuput oil were analyzed by qRT-PCR. The MICs and MFCs of Thai Cajuput oil ranged from 0.31 to 1.25 *μ*l/ml and 0.63 to 1.25 *μ*l/ml, respectively, and its activity was defined as fungicidal activity. The MICs of the combination of Thai Cajuput oil and fluconazole were much lower than the MICs of the individual drugs. Interestingly, sub-MICs of Thai Cajuput oil significantly reduced the *MDR*1 expression level in resistant strains (*P* < 0.05). Our study suggests that Thai Cajuput oil can be used to create new potential combination therapies to combat the antifungal resistance of *C. albicans*.

## 1. Introduction

Candidiasis is one of the most important fungal infections caused by *Candida* species; these infections pose a challenging threat to human health and affect millions of individuals. More than 17 species of *Candida* are responsible for causing both superficial and life-threatening systemic mycoses, and of these, *Candida albicans* is a major fungal pathogen accounting for invasive candidiasis and is the most common cause of nosocomial infections. *C. albicans* is a normally dimorphic commensal organism and is part of the normal flora of healthy people. However, *C. albicans* can undergo a transition from a commensal to pathogenic form in immunocompromised hosts/normal individuals and function as an opportunistic pathogen. *C. albicans* has a unique ability to grow in a variety of morphological forms (yeast-like, hyphae, pseudohyphae, and chlamydospores), which can serve as key virulence factors. In addition, the growth of *Candida* biofilms on medical devices has emerged as a major challenge in hospital settings, leading to secondary fungal infections and resulting in morbidity and mortality of immunocompromised individuals (including HIV-infected patients, transplant recipients, chemotherapy patients, diabetes patients, and low-birth-weight infants) [1, 2].

With the improvements made in the healthcare system worldwide, the numbers of elderly people and immunocompromised patients have dramatically increased for several reasons (including immunosuppressive treatments, long-term catheterization, use of broad-spectrum antibiotics, and longer survival of immunologically compromised individuals), resulting in an increase in candidiasis prevalence, emergence of drug resistance, lower availability of antifungal drugs, failure of treatment, and recalcitrant infections [3, 4]. In 2019, the Centers for Disease Control and Prevention (CDC) reported that *C. albicans* is the most common cause of bloodstream infections and that antifungal resistance is a particular problem. CDC surveillance data indicate that the proportion of *Candida* isolates that are resistant to fluconazole has remained fairly constant over the past 20 years [5].

Among the different antifungals available, fungistatic azoles are widely used to treat *Candida* infections due to their high efficacy, reduced toxicity, and low cost. The emergence of azole-resistant *C. albicans* strains is associated with the widespread and prolonged use of azoles, especially fluconazole (FLC) [6, 7]. A common mechanism of fluconazole resistance in *C. albicans* is the failure of cells to accumulate the drug due to increased expression of certain proteins encoded by the *CDR*1, *CDR*2, and *MDR*1 genes. The *CDR*1 and *CDR*2 genes encode proteins that belong to the superfamily of ATP-binding cassette (ABC) transporters. The *MDR*1 gene encodes a protein that belongs to the major facilitator superfamily (MFS) transporters. Overexpression of these transporters plays an important role in fluconazole resistance in *C. albicans* due to increased fluconazole efflux [8]. Several studies have demonstrated enhanced susceptibility of *C. albicans* to fluconazole as a result of the disruption of the *CDR*1, *CDR*2, and *MDR*1 genes [9, 10].

Therefore, there is a constant demand for the development of a new generation of antifungal agents with superior antifungal properties and minimal host toxicity. The use of natural products and supplements has increased around the world, and scientists have been interested in studying plant products as antimicrobial or antifungal agents [11]. In the present study, we investigated Thai Cajuput oil from *Melaleuca cajuputi* Powell. (Cajuput tree), which belongs to the Myrtaceae family and exclusively grows in Southern and Eastern Thailand. Myrtaceae is one of the most important essential oil-producing families and has diverse bioactivities, such as antibacterial, antifungal, antioxidant, insecticidal, and antiviral activities. Cajuput oil from *M. cajuputi* has been used in medicine since the eighteenth century as an antiseptic agent, and its effect is comparable to that of tea tree oil from *M. alternifolia* [12]. At a concentration of 0.4–0.6%, Cajuput oil inhibits the growth of yeast, such as *C. albicans, C. vaginalis,* and *C. glabrata*, and mold, such as *Aspergillus niger* and *Penicillium notatum* [13, 14]. These findings support the theory that Thai Cajuput oil may inhibit the growth of *C. albicans* and reduce the expression of genes that play an important role in fluconazole resistance in *C. albicans*.

In this study, we first investigated efflux-pump genes (*CDR*1, *CDR*2, and *MDR*1) in fluconazole-resistant *C. albicans* clinical isolates and evaluated the in vitro antifungal activity of Thai Cajuput oil against resistant strains. In addition, we performed a checkerboard assay to investigate the antifungal effects of Thai Cajuput oil combined with FLC against resistant strains and to identify whether the combinations could also reverse one of the common resistance mechanisms of increased drug efflux pumps. We conducted qRT-PCR to assess the effect of Thai Cajuput oil on efflux-pump gene expression. The explanation of such mechanisms could help in the development of new therapeutic strategies, new alternative therapies, and the subsequent reversion of antifungal resistance in *Candida* species.

## 2. Materials and Methods

### 2.1. Strains, Media, and Growth Conditions

Sixteen FLC-resistant *C. albicans* clinical strains and *C. albicans* ATCC 90028 were used in this study. All isolates originated from the collection of the Department of Microbiology, Faculty of Science, Burapha University, Chonburi Province, Thailand. Clinical strains were isolated from sputum and urine of hospitalized patients with mycoses in Somdej Phra Boromma Ratchathewi Na Si Racha Hospital, Chonburi Province, Thailand. The isolates were periodically identified by phenotypic techniques (microscopic morphology and macroscopic morphology on Sabouraud dextrose agar and chromogenic *Candida* agar) and confirmed by conventional PCR assay. The strains were maintained in 30% glycerol at −80°C and subcultured at least twice on Sabouraud dextrose agar (HiMedia Laboratories, India) at 35°C. Their FLC susceptibilities were determined by the broth microdilution method according to the Clinical and Laboratory Standards Institute (CLSI) documents M27-A3 [15] and M60 [16] with *C. albicans* ATCC 90028 as the quality control strain. FLC was purchased from Sigma-Aldrich, US. The stock solution of FLC was prepared according to the manufacturer's instructions. RPMI-1640 (Gibco, Carlsbad, CA) buffered with MOPS (AppliChem GmbH, Germany) and supplemented with 0.2% glucose was used as the diluent medium for drug and strains.

### 2.2. Plant Material and Essential Oil Extraction

Leaves and twigs of *Melaleuca cajuputi* Powell (Cajuput tree) were collected from the Community Forest in Tha Chang District (The Chaipattana Foundation), Chanthaburi Province, Thailand, in February 2018. Fresh leaves and twigs were harvested at random from 15–20 trees in a forest. After the natural drying of samples, 6050 g of samples was collected. Essential oils were extracted by hydrodistillation [17]. Oil was extracted as follows: an extraction temperature of 65–70°C and an extraction time of 48–72 h. The resultant yield of oil was 0.35%. Oil was analyzed by gas chromatography-mass spectrometry (GC-MS), and the six major constituents were 1,8-naphthyridine derivatives (10.46%), alpha-pyrone (10.11%), terpinolene (9.26%), gamma-terpinene (8.00%), beta-caryophyllene (6.36%), and beta-elemene (5.09%), defined as a component present at 5% or greater of the total composition. However, the oil displayed similar major components to some studies of *Melaleuca cajuputi* Powell essential oil from pure leaves were grown in different parts of Thailand, but differences in their quantity [18–20]. It was preserved at 4°C in a dark brown vial in the dark until further analysis [21]. The extracted oil should have been dissolved into 100% dimethyl sulfoxide (DMSO) to continue all the experiments conducted in this study, following which they were serial diluted in a test medium to reach the concentrations to be tested. The final concentration of DMSO in the assays did not exceed 1%, which did not affect fungal growth [22].

### 2.3. DNA Extraction and Amplification

To screen the efflux-pump genes (*CDR*1, *CDR*2, and *MDR*1) among resistant strains, genomic DNA was extracted from sixteen resistant strains using organic extraction (phenol: chloroform) as described by Javadi et al. [23] with modifications. Briefly, the cell suspension (10^7^ cells/ml) was lysed in lysis buffer combined with a freeze-thaw process, treated with RNase A (Invitrogen™, Thermo Fisher Scientific, Inc., US) to inactivate cellular RNase, treated with phenol/chloroform/isoamyl alcohol (Research Organics, Inc., USA) to separate gDNA from cell debris, precipitated with ethanol and eluted in TE buffer. After extraction, DNA was quantified using a NanoDrop One^C^ (Thermo Fisher Scientific Inc., US). The obtained DNA samples were subjected to PCR amplification. PCR was prepared in a total volume of 20 *μ*l, which included 10 ng of DNA template, 1x FIREPol® master mix (FIREPol^®^ DNA polymerase, 1x reaction buffer B, 2.5 mM MgCl_2_, 200 *µ*M dNTPs) and gene-specific primers (LGC, Biosearch Technologies, Inc., listed in Table 1). Amplification was carried out in a Mastercycler^®^ nexus (Eppendorf AG, Germany) using conditions modified from Ricardo et al. [10]. Reactions were performed as follows: initial denaturation at 95°C for 4 mins, followed by 35 cycles of denaturation at 95°C for 20 secs, annealing at 55–58°C for 1 min (Table 1) and extension at 72°C for 20 secs, and a final extension at 72°C for 4 mins. The appropriate positive control and no template control were included in each experiment. The amplicons were separated by electrophoresis on a 2% agarose gel, analyzed and visualized under blue light using an Omega Fluor™ Plus (Gel Company, Inc., USA).

### 2.4. Antifungal Activity of Essential Oil

The broth macrodilution method was used to determine the minimum inhibitory concentration (MIC) and minimum fungicidal concentration (MFC) of the Thai Cajuput oil against resistant strains according to the CLSI documents M27-A3 [15] and M60 [16] with *C. albicans* ATCC 90028 as the quality control strain. In brief, cell suspensions of sixteen resistant strains (0.5–2.5 × 10^3^ cells/ml) in an RPMI-1640 medium (w/MOPS + 0.2% glucose) were added to serial tubes cultured with different concentrations of Thai Cajuput oil and were then incubated at 37°C for 48 h. The final concentrations of Thai Cajuput oil were two-fold serially diluted in a test medium ranging from 0.04 to 5 *μ*l/mL (0.04, 0.08, 0.16, 0.31, 0.63, 1.25, 2.5, and 5 *μ*l/ml), and the final concentration of DMSO did not exceed 1%. The growth control contained yeast cells and broth medium with no essential oil. The solvent control contained yeast cells and a broth medium with 1% DMSO. The purity control contained only a broth medium. To decide and confirm the primary decision regarding the MIC and MFC of Thai Cajuput oil, 10 *μ*l of broth from the first tube that showed no clear growth and the two successive clear tubes were dropped onto SDA plates. Then, the inoculated plates were incubated at 37°C for 24 h. The MIC was defined as the lowest concentration of Thai Cajuput oil that inhibited visible growth of *C. albicans* isolates within the tube. On the other hand, the MFC was defined as the lowest concentration of Thai Cajuput oil that completely inhibited the growth of *C. albicans* isolates on SDA plates [24]. The experiment was repeated three times, and the average MICs and MFCs were recorded. The MFC/MIC ratio was calculated to determine antifungal activity, and a substance was defined as having fungistatic activity when the MFC/MIC ratio ≥4 and as having fungicidal activity when the MFC/MIC ratio <4 [25].

### 2.5. Checkerboard Synergy Analysis

The effect of FLC combined with Thai Cajuput oil was evaluated using the checkerboard method with slight modifications [26]. Concisely, four resistant strains (S7/1, U6/2, U15/1, and U8/1) that showed different efflux-pump gene patterns were used, and cell suspensions of these strains (0.5–2.5 × 10^3^ cells/ml) in RPMI-1640 medium (w/MOPS + 0.2% glucose) were added to a 96-well plate cultured with different concentrations of FLC + Thai Cajuput oil and were then incubated at 37°C for 48 h. The final concentrations of FLC were two-fold serially diluted in a test medium ranging from 0.125 to 64 mg/mL (0.125, 0.25, 0.5, 1, 2, 4, 8, 16, 32, and 64 *μ*g/mL), the final concentration of Thai Cajuput oil was two-fold serially diluted in a test medium ranging from 0.04 to 1.25 *μ*l/mL (0.04, 0.08, 0.16, 0.31, 0.63, and 1.25 *μ*l/mL), and the final concentration of DMSO did not exceed 1%. The growth control contained yeast cells and a broth medium with no drug and essential oil. The solvent control contained yeast cells and a broth medium with 1% DMSO. The purity control contained only a broth medium. The experiment was repeated three times, and drug interactions were interpreted by the fractional inhibitory concentration index (FICI) model. The FICI was calculated as the sum of the FICs of either drug (MIC_FLC + Cajuput oil_/MIC_FLC_ + MIC_Cajuput oil + FLC_/MIC_Cajuput oil_). An FICI ≤0.5 represents synergistic activity; >0.5 and ≤1 additive activity; >1 and ≤4 indifferent activity; and >4 antagonistic activity [27].

### 2.6. Gene Expression Analysis

To compare the changes in the expression of the efflux-pump genes (*CDR*1, *CDR*2, and *MDR*1), total RNA was extracted from resistant strains before and after treatment with Thai Cajuput oil. Four resistant strains (S7/1, U6/2, U15/1, and U8/1) that showed different efflux-pump gene patterns were selected and incubated in SDB at 37°C for 24 h with continuous shaking at 240 rpm. The cell suspensions (10^7^ cells/ml) were treated with ½MIC, ¼MIC, and ⅛MIC of Thai Cajuput oil and 1% DMSO as solvent control in SDB at 37°C for 90 min with continuous shaking at 240 rpm. Cultures without Thai Cajuput oil served as the untreated. Cells were then harvested for RNA extraction, and total RNA was isolated using TRIsure™ reagent (Bioline, UK) following the manufacturer's instructions. The RNA samples were quantified using a NanoDrop One^C^ (Thermo Fisher Scientific Inc., US).

One-step real-time reverse transcription PCR (qRT-PCR) was performed using the One-step BrightGreen qRT-PCR Kit (Applied Biological Materials, Inc., Canada) following the manufacturer's instructions. RT-PCR was performed in a total volume of 10 *μ*l, which included 5 ng of total RNA, 1X BrightGreen qPCR master mix, 1X qRT-PCR enzyme mix, and gene-specific primers (LGC, Biosearch Technologies, Inc., listed in Table 1). Amplifications were carried out in the CFX96 Touch™ Real-Time PCR Detection System (Bio-Rad Laboratories, Inc., USA). The following cycling protocol was used: cDNA synthesis at 42°C for 15 mins, predenaturation at 95°C for 4 mins, followed by 35 cycles of denaturation at 95°C for 20 secs, and annealing/elongation at 55–58°C for 1 min (Table 1). The appropriate positive control and no template control were included in each experiment. To verify the specificity of the amplicon, the products were evaluated via melting point analysis conducted directly after amplification to determine the dissociation of the PCR products from 65°C to 95°C. The threshold cycle (*Ct*) values were automatically determined by CFX Manager™ Software (Bio-Rad Laboratories Inc., USA) using the default parameters. The *Ct* values are defined as the cycle number when the fluorescence signal of a PCR product can be detected above the background signal. The *Ct* value is a relative measure of the concentration of target in the PCR reaction.

The expression levels of the efflux-pump genes (*CDR*1, *CDR*2, and *MDR*1) were evaluated using the 2^−∆∆*Ct*^ method, where cycle threshold (*Ct*) is the abbreviation for quantification cycle, formerly known as cycle quantification (*Cq*). The qRT-PCR reactions were performed in duplicate, and *Ct* values were averaged according to the Minimum Information for Publication of Quantitative Real-Time PCR Experiments (MIQE) guidelines [28]. Essentially, all data were normalized to the *ACT*1 housekeeping gene as the internal reference gene and were compared to an untreated calibrator sample. The data are presented as the fold change of gene expression.

### 2.7. Statistical Analysis

The broth macrodilution method and the checkerboard assay were performed in three independent experiments, and the results are reported relative to the control. Statistical analyses were performed using the statistical package for social sciences (SPSS), version 22.0 release for Microsoft Windows (SPSS, Inc., Chicago). The expression levels of the efflux genes before and after treatment were compared using the paired *t-*test, in which a *p* value of less than 0.05 was considered statistically significant.

## 3. Results

### 3.1. Efflux-Pump Gene Patterns among Resistant Strains

**Figure 1:**
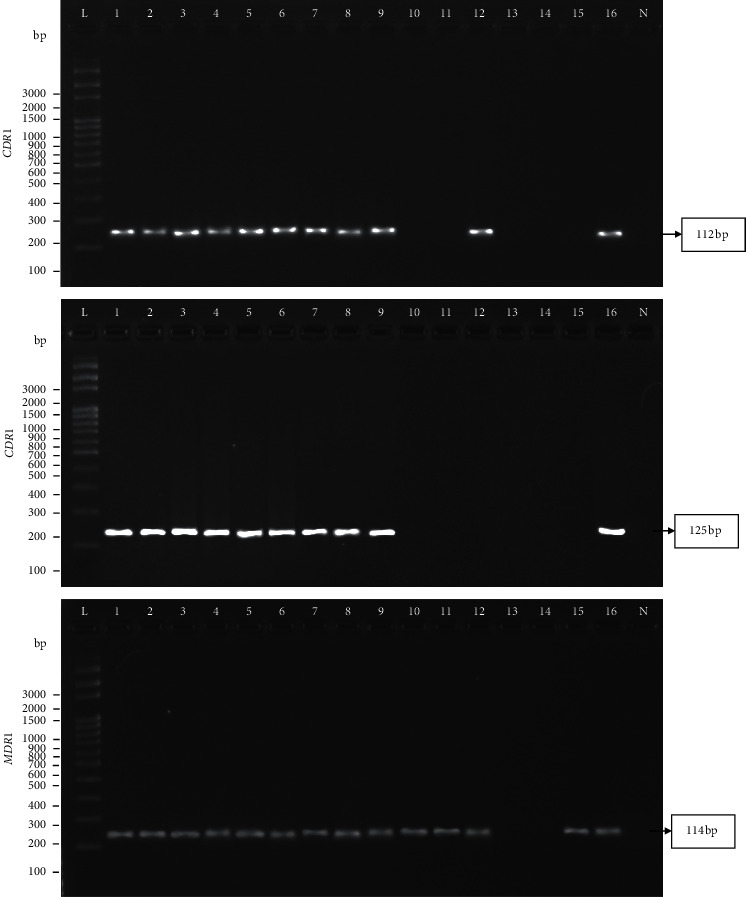
PCR products of the *CDR*1, *CDR*2, and *MDR*1 genes among sixteen resistant strains. Lanes L: 100-bp DNA ladder as the molecular size marker; Lanes 1: S7/1 strain as the positive control (+ve); Lanes 2–16: S8/1, S9/1, S10/1, S14/1, S15/1, U6/1, U6/2, U7/1, U8/1, U11/1, U15/1, U15/2, U19/1, U21/1, and U25/1 strains, respectively. Lanes N: no template control (−ve).

According to efflux-pump gene screening among sixteen resistant strains by PCR, efflux-pump genes were detected in fourteen resistant strains (87.5%) but not in two resistant strains (12.5%). The PCR products of the *CDR*1, *CDR*2, and *MDR*1 genes after separation by electrophoresis on an agarose gel are shown in Figure 1. The presence of the *MDR*1 gene showed the highest frequency among the fourteen resistant strains. The frequencies of the *CDR*1, *CDR*2, and *MDR*1 genes were 68.75%, 62.5%, and 87.5%, respectively. The efflux-pump genes were distributed in three distinct patterns: pattern A was *CDR*1*-*, *CDR*2*-*, and *MDR*1*-*positive (*n* = 10); pattern B was *CDR*1*-* and *MDR*1*-*positive (*n* = 1); and pattern C was only *MDR*1*-*positive (*n* = 3), as presented in Table 2.

### 3.2. Antifungal Activity of Thai Cajuput Oil

The antifungal activity of Thai Cajuput oil against the fourteen resistant strains was evaluated using the broth macrodilution method. The mean of three independent experiments was determined and reported as MICs, MFCs, and MFC/MIC ratios in Table 2. Thai Cajuput oil inhibited the growth of all resistant strains. The MICs and MFCs ranged from 0.31 to 1.25 *μ*l/ml and 0.63 to 1.25 *μ*l/ml, respectively. According to the MFC/MIC ratio calculation, the ratios were within the range of 1 to 2, and the antifungal activity of Thai Cajuput oil was defined as fungicidal activity.

### 3.3. Synergism of Thai Cajuput Oil and Fluconazole

In the checkerboard synergy analysis, the MICs of Thai Cajuput oil and FLC were assessed alone and in combination against four resistant strains that had different efflux-pump gene patterns, which each patterns may have different expression level of efflux-pump genes and showed various FLC resistance level. The isolate with high FLC resistance level could be detected only one, two, or three efflux-pump genes. Four isolates were chosen, at least one isolate from each pattern, to comprehensively perform the synergy analysis. The MICs of FLC alone in the S7/1, U6/2, U15/1, and U8/1 strains were 8, 64, 64, and 16 *μ*g/ml, respectively. The MIC of Cajuput oil alone in the four resistant strains was 1.25 *μ*l/ml. When Cajuput oil and FLC were combined, the MICs of the combination were much lower than the MICs of the individual drugs. In the presence of low Cajuput oil concentrations, the MICs of FLC decreased by 1 to 64 times. For Cajuput oil, the MICs decreased by 8 to 32 times. According to the FICI calculation, Cajuput oil and FLC exhibited a synergistic effect in the S7/1, U6/2, and U15/1 strains, which had FICIs of 0.189, 0.159, and 0.144, respectively. The U8/1 strain exhibited an indifferent effect, with an FICI of 1.032, as presented in Table 3.

### 3.4. Efflux-Pump Genes Expression

**Figure 2:**
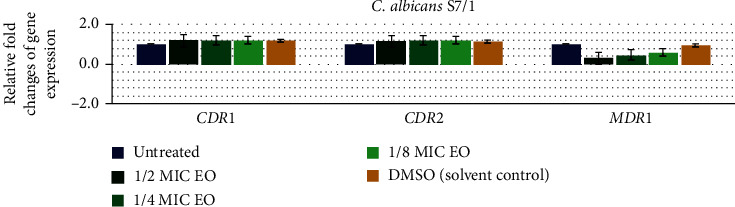
Relative expression of the efflux-pump genes in the S7/1 strain. The strain was treated with ½MIC, ¼MIC, and ⅛MIC of Cajuput oil for 90 mins. Using the 2^−∆∆Ct^ method, data are presented as the fold change in gene expression normalized to an endogenous reference gene (*ACT*1) and compared to the untreated control. Values represent the mean ± SD from two independent experiments. ^*∗*^Statistically significant differences between values (*p* < 0.05).

**Figure 3:**
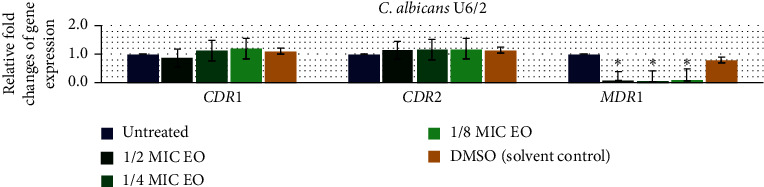
Relative expression of the efflux-pump genes in the U6/2 strain. The strain was treated with ½MIC, ¼MIC, and ⅛MIC of Cajuput oil for 90 mins. Using the 2^−∆∆Ct^ method, data are presented as the fold change in gene expression normalized to an endogenous reference gene (*ACT*1) and compared to the untreated control. Values represent the mean ± SD from two independent experiments. ^*∗*^Statistically significant differences between values (*p* < 0.05).

**Figure 4:**
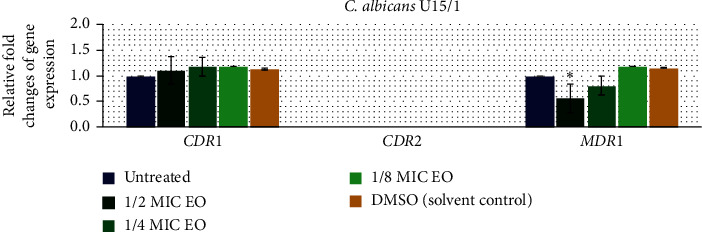
Relative expression of efflux-pump genes in the U15/1 strain. The strain was treated with ½MIC, ¼MIC, and ⅛MIC of Cajuput oil for 90 mins. Using the 2^−∆∆Ct^ method, data are presented as the fold change in gene expression normalized to an endogenous reference gene (*ACT*1) and compared to the untreated control. Values represent the mean ± SD from two independent experiments. ^*∗*^Statistically significant differences between values (*p* < 0.05).

**Figure 5:**
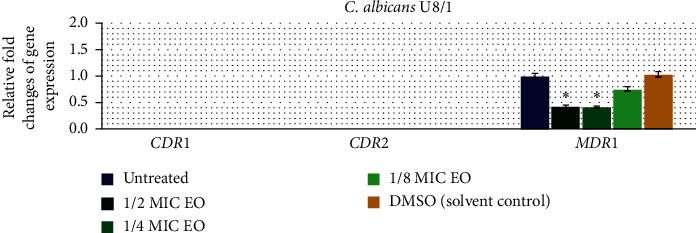
Relative expression of efflux-pump genes in the U8/1 strain. The strain was treated with ½MIC, ¼MIC, and ⅛MIC of Cajuput oil for 90 mins. Using the 2^−∆∆Ct^ method, data are presented as the fold change in gene expression normalized to an endogenous reference gene (*ACT*1) and compared to the untreated control. Values represent the mean ± SD from two independent experiments. ^*∗*^Statistically significant differences between values (*p* < 0.05).

To investigate the effect of Thai Cajuput oil on four resistant strains with different efflux-pump gene patterns, the expression levels of the efflux-pump genes were evaluated using qRT-PCR. Quantification data for all the genes were normalized to the reference gene and compared to the untreated control. There were no primer dimers or nonspecific amplification products according to the melting curves and melting peaks of all the tested genes. The relative fold changes in *CDR*1, *CDR*2, and *MDR*1 gene expression in the S7/1, U6/2, U15/1, and U8/1 strains are shown in Figures 2–5, respectively. For all tested strains, there was no significant difference in the *CDR*1 and *CDR*2 expression levels among treatments (*p* > 0.05); however, the *MDR*1 expression levels among treatments with ½MIC, ¼MIC, and ⅛MIC of Cajuput oil decreased. Concisely, subinhibitory concentrations of Cajuput oil (½MIC) only significantly reduced the *MDR*1 expression level in resistant strains (*p* > 0.05).

## 4. Discussion

Fluconazole-resistant *C. albicans* is responsible for the most prevalent nosocomial fungal infections and has resulted in many clinical treatment failures. Different mechanisms may be expressed, allowing organisms to elude the effects of antifungals. One of the most important molecular mechanisms causing azole resistance in *C. albicans* is the increased efflux of a drug, mostly mediated by the ATP-binding cassette (ABC) and major facilitator superfamily (MFS) transporters [29]. The emergence of fungal resistance to antifungal agents has been rapidly increasing, but the rate of new antifungal production is insufficient to meet the current need [30]. This situation indicates the importance of developing new promising antifungal agents or exciting the future of antifungal therapy. The goal of this study was to investigate the effect of *Melaleuca cajuputi* Powell (Thai Cajuput oil) on efflux-pump gene (*CDR*1, *CDR*2, and *MDR*1) expression in fluconazole-resistant *C. albicans* clinical isolates using qRT-PCR.

In this investigation, we first identified genes encoding drug efflux pumps (*CDR*1, *CDR*2, and *MDR*1) among sixteen resistant strains. All the efflux-pump genes were detected in fourteen of sixteen resistant strains, and the presence of the *MDR*1 gene showed the highest frequency among resistant strains. The frequencies of the *CDR*1, *CDR*2, and *MDR*1 genes were 68.75%, 62.5%, and 87.5%, respectively. Comparably, Perea et al. observed efflux-pump genes (*CDR* and *MDR*1) in 86% of all azole-resistant isolates, and overexpression of the *CDR* and *MDR*1 genes was detected in 83% and 67%, respectively, of azole-resistant isolates [8]. Many studies have confirmed that the nature of azole resistance is multifactorial, with a predominance of the overexpression of genes encoding drug efflux pumps [31–33].

Thai Cajuput oil is obtained from leaves and twigs of *Melaleuca cajuputi* Powell. (Cajuput tree), which belongs to the Myrtaceae family. Although essential oils from Myrtaceae species have diverse bioactivities, reports on the antifungal activity of essential oils from *M. cajuputi* are limited. The results of our study also demonstrated the strong antifungal activity of Thai Cajuput oil against fourteen resistant strains that carried efflux-pump genes. The MICs and MFCs ranged from 0.31 to 1.25 *μ*l/ml and 0.63 to 1.25 *μ*l/ml, respectively. The antifungal activity of Thai Cajuput oil was defined as fungicidal activity. Similar results have been reported the same genus in the Myrtaceae family: the MICs of *M. alternifolia* (tea tree oil) against FLC-resistant *C. albicans* clinical isolates are 2.5–5 *μ*l/ml as described by Mondello et al. [34] and the MICs and MFCs of *M. alternifolia* oil against *C. albicans* ATCC 90028 are 2.5 *μ*l/ml as described by Francisconi et al. [35]. However, the antifungal activity of Thai Cajuput oil could be attributed to its complex mixture of biologically active compounds, which greatly differs according to the chemotype of the plant and the place from which the plant was obtained. In this work, essential oil from *M. cajuputi* Powell contained six important components, including 1,8-naphthyridine derivatives, alpha-pyrone, terpinolene, gamma-terpinene, beta-caryophyllene, and beta-elemene. Some studies have indicated that compounds present in spices may also play a major role in their antifungal effects. Acosta et al. described that the potential antifungal activity of 1,8-naphthyridine derivatives is related to their hydrophobicity, which disrupts the membrane integrity of *C. albicans* and *Cryptococcus neoformans* cells [36]. Similarly, Pereira et al. indicated that beta-caryophyllene may be absorbed by the fungal cell membrane and act as an antifungal agent by releasing lipophilic drugs [37]. Additionally, the antifungal effects of terpinolene and gamma-terpinene have been shown in another study [38]. These findings suggest that the effect of Thai Cajuput oil may be related to these components.

Regarding the synergism of Thai Cajuput oil and FLC against four resistant strains that had different efflux-pump gene patterns, we found that the MICs of drug combinations were much lower than the MICs of the individual drugs. In the presence of low concentration of Cajuput oil, the MICs of FLC decreased by 8 to 64 times. For Cajuput oil, the MICs decreased by 8 to 16 times. Cajuput oil and FLC exhibited a synergistic effect in three resistant strains with FICIs of 0.144 to 0.189. Similar results indicate that *M. leucadendra* oil and FLC exhibit a synergistic effect in *C. albicans*, with an FICI of 0.35; when they were combined, the MICs of FLC decreased by 4 times and the MICs of *M. leucadendra* oil decreased by 10 times, as described by Zhang et al. [39]. The synergism of Thai Cajuput oil and FLC suggests that essential oil may play a role in reducing drug resistance in *C. albicans* clinical isolates. Evaluating the expression of genes that are responsible for drug resistance in *C. albicans* is the most accurate method to prove this hypothesis.

According to qRT-PCR, our findings revealed that the expression levels of *CDR*1 and *CDR*2 were not significantly different among treatments (*p* > 0.05), while the *MDR*1 expression levels upon treatment with ½MIC, ¼MIC, and ⅛MIC of Cajuput oil were decreased. The subinhibitory concentrations of Cajuput oil (½MIC) only significantly reduced the *MDR*1 expression level in resistant strains (*p* < 0.05). It is known that the overexpression of the *MDR*1 gene is the main cause of the prevalence of FLC-resistant *C. albicans*, which effectively reduces the intracellular concentration of antifungal agents and ultimately makes FLC ineffective. Ahmad et al. showed that the inhibition of efflux-pump genes (*CDR*1 and *MDR*1) in FLC-resistant *Candida* spp. by two monoterpenes is a feasible strategy to overcome clinical antifungal resistance [40]. Similarly, Morschhäuser J. demonstrated that disruption of the *MDR*1 gene in the *C. albicans* strain, in which it was strongly expressed, resulted in increased susceptibility of the mutants to several antifungal drugs [41]. The impact of this report indicates for the first time that Thai Cajuput oil was identified as an inhibitor of FLC-resistant *C. albicans* clinical isolates which reduced the expression level of the *MDR*1 gene. In addition, it is interesting to study further on the point of effect of Thai Cajuput oil inhibits *MDR*1 expression by repressing transcription factors such as *Mrr*1. The great influence of this study indicates that Thai Cajuput oil can be successfully employed as a good candidate for the inhibition of *C. albicans* growth and may be a potential source of natural antifungal agents to battle resistant *Candida* infections.

## 5. Conclusions

In summary, Thai Cajuput oil is widely distributed in Eastern Thailand. Leaves and twigs of *M. cajuputi* Powell were found to have potent antifungal activity against FLC-resistant *C. albicans* clinical isolates. Thai Cajuput oil was able to reduce the MIC of fluconazole and reduce the expression level of *MDR*1, an important gene that plays a role in resistance to azole drugs in *C. albicans*. These results are of considerable importance due to the increasing resistance of emerging fungal species to available drugs used to treat a variety of fungal infections and for the exploration of potential alternative therapeutic sources for multidrug resistant therapy.

## Figures and Tables

**Table 1:** Primers for the *CDR*1, *CDR*2, *MDR*1, and *ACT*1 genes for PCR and qRT-PCR [10].

Genes	Primers	Sequences	Primer concentration (*μ*M)	Annealing temperature (°C)	Amplicon size (bp)	GenBank accession no.
*CDR*1	Forward	5'-TGCCAAACAATCCAACAA-3'	0.5	58	112	X77589
	Reverse	5'-CGACGGATCACCTTTCATACGA-3'	0.5			

*CDR*2	Forward	5'-AAGGTTTTGATGCTACTGC-3'	0.5	55	125	U63812
	Reverse	5'-GTCGGACATGTGGCTCAAA-3'	0.5			

*MDR*1	Forward	5'-GTGTTGGCCCATTGGTTTTCAGTC-3'	0.7	58	114	X53823
	Reverse	5'-CCAAAGCAGTGGGGATTTGTAG-3'	0.7			

*ACT*1	Forward	5'-AAGAATTGATTTGGCTGGTAGAGA-3'	0.5	58	179	X16377
	Reverse	5'-TGGCAGAAGATTGAGAAGAAGTTT-3'	0.5			

**Table 2:** Efflux-pump gene patterns among resistant strains and antifungal activity of Thai Cajuput oil.

Efflux-pump gene patterns	Resistant strains	MICFLC^a^ (*μ*g/ml)	MIC_Cajuput oil_^a^ (*μ*l/ml)	MFC_Cajuput oil_^a^(*μ*l/ml)	MFC/MIC ratio	Antifungal activity
A: *CDR*1, *CDR*2, and *MDR*1	S7/1	8	1.25	1.25	1	Fungicidal
	S8/1	16	1.25	1.25	1	Fungicidal
	S9/1	≥64	1.25	1.25	1	Fungicidal
	S10/1	≥64	1.25	1.25	1	Fungicidal
	S14/1	8	1.25	1.25	1	Fungicidal
	S15/1	≥64	1.25	1.25	1	Fungicidal
	U6/1	≥64	1.25	1.25	1	Fungicidal
	U6/2	≥64	1.25	1.25	1	Fungicidal
	U7/1	≥64	1.25	1.25	1	Fungicidal
	U25/1	≥64	1.25	1.25	1	Fungicidal

B: *CDR*1 and *MDR*1	U15/1	≥64	1.25	1.25	1	Fungicidal

C: *MDR*1	U8/1	16	1.25	1.25	1	Fungicidal
	U11/1	16	0.31	0.63	2	Fungicidal
	U21/1	16	0.63	1.25	2	Fungicidal

^a^Data are presented as the mean of three independent experiments and presented relative to control.

**Table 3:** Efflux-pump gene patterns among resistant strains and antifungal activity of Thai Cajuput oil.

Efflux-pump gene patterns	Resistant strains	Drugs	MIC alone^a^	MIC combination^a^	FIC	FICI	Interpretation
A: *CDR*1, *CDR*2, and *MDR*1	S7/1	FLC	8	1	0.125	0.189	Synergistic
		Cajuput oil	1.25	0.08	0.064		
	U6/2	FLC	64	2	0.031	0.159	Synergistic
		Cajuput oil	1.25	0.16	0.128		

B: *CDR*1 and *MDR*1	U15/1	FLC	64	1	0.016	0.144	Synergistic
		Cajuput oil	1.25	0.16	0.128		

C: *MDR*1	U8/1	FLC	16	16	1	1.032	Indifferent
		Cajuput oil	1.25	0.04	0.032		

^a^Data are presented as the mean of three independent experiments and presented relative to control.

## Data Availability

Data used to support the findings of this study are included within the article and also available from the corresponding author upon request.
